# Next-Generation Sequencing and Proteomics of Cerebrospinal Fluid From COVID-19 Patients With Neurological Manifestations

**DOI:** 10.3389/fimmu.2021.782731

**Published:** 2021-12-09

**Authors:** Haijun Wang, Zili Zhang, Junfen Zhou, Shuqing Han, Zhenyu Kang, Haoyu Chuang, Heng Fan, Hongyang Zhao, Lin Wang, Yunjia Ning, Alexey Sarapultsev, Willis X. Li, Jinghong Li, Zhicheng Lin, Shanshan Luo, Nian Xiong, Desheng Hu

**Affiliations:** ^1^ Department of Neurosurgery, Union Hospital, Tongji Medical College, Huazhong University of Science and Technology, Wuhan, China; ^2^ Department of Integrated Traditional Chinese and Western Medicine, Union Hospital, Tongji Medical College, Huazhong University of Science and Technology, Wuhan, China; ^3^ Department of Nuclear Medicine, The Central Hospital of Wuhan, Tongji Medical College, Huazhong University of Science and Technology, Wuhan, China; ^4^ Department of Neurosurgery, Wuhan Red Cross Hospital, Wuhan, China; ^5^ Department of Neurosurgery, Tainan Municipal An-Nan Hospital, Tainan, Taiwan; ^6^ Department of Clinical Laboratory, Union Hospital, Tongji Medical College, Huazhong University of Science and Technology, Wuhan, China; ^7^ Wuhan Institute of Virology, Chinese Academy of Sciences, Wuhan, China; ^8^ Institute of Immunology and Physiology, Ural Branch of the Russian Academy of Science, Ekaterinburg, Russia; ^9^ Department of Medicine, University of California San Diego, La Jolla, CA, United States; ^10^ Laboratory of Psychiatric Neurogenomics, McLean Hospital, Harvard Medical School, Belmont, MA, United States; ^11^ Institute of Hematology, Union Hospital, Tongji Medical College, Huazhong University of Science and Technology, Wuhan, China; ^12^ Department of Neurology, Union Hospital, Tongji Medical College, Huazhong University of Science and Technology, Wuhan, China; ^13^ Department of Neurology, Wuhan Red Cross Hospital, Wuhan, China

**Keywords:** SARS-CoV-2, neurological manifestations, proteomics, next-generation sequencing, complement system

## Abstract

The SARS-CoV-2 and its variants are still hitting the world. Ever since the outbreak, neurological involvements as headache, ageusia, and anosmia in COVID-19 patients have been emphasized and reported. But the pathogenesis of these new-onset neurological manifestations in COVID-19 patients is still obscure and controversial. As difficulty always lay in the diagnosis of neurological infection, current reports to validate the presence of SARS-CoV-2 in cerebrospinal fluid (CSF) almost relied on the basic methods and warranted improvement. Here we reported a case series of 8 patients with prominent new-onset neurological manifestations, who were screened out from a patch of 304 COVID-19 confirmed patients. Next-generation sequencing (NGS) and proteomics were conducted in the simultaneously obtained CSF and serum samples of the selected patients, with three non-COVID-19 patients with matched demographic features used as the controls for proteomic analysis. SARS-CoV-2 RNA was detected in the CSF of four COVID-19 patients and was suspicious in the rest four remaining patients by NGS, but was negative in all serum samples. Proteomic analysis revealed that 185 and 59 proteins were differentially expressed in CSF and serum samples, respectively, and that only 20 proteins were shared, indicating that the proteomic changes in CSF were highly specific. Further proteomic annotation highlighted the involvement of complement system, PI3K-Akt signaling pathway, enhanced cellular interaction, and macrophages in the CSF proteomic alterations. This study, equipped with NGS and proteomics, reported a high detection rate of SARS-CoV-2 in the CSF of COVID-19 patients and the proteomic alteration of CSF, which would provide insights into understanding the pathological mechanism of SARS-CoV-2 CNS infection.

## Introduction

The coronavirus disease 2019 (COVID-19) pandemic, caused by the severe acute respiratory syndrome coronavirus-2 (SARS-CoV-2), have posed great challenge to the world and is still raging without signs of vanishment, as the spread of the more contagious Delta variants. By November 11, 2021, there have been 251,266,207 confirmed cases of COVID-19 with 5,070,244 deaths (1).

For symptomatic COVID-19, the clinical manifestations range from mild flu-like symptoms to severe respiratory failure. As evidences accumulates to depict the comprehensive features of SARS-CoV-2 infection ever since the outbreak, neurological involvement is found to be quite prevalent, which bring into question the neurotropism of SARS-CoV-2. Mao et al. reported that more than a third of COVID-19 patients experienced neurological manifestations, based on their clinical signs and symptoms (2). A study from France reported the neurological features of 58 COVID-19 patients with encephalopathy and corticospinal tract damage signs (3). And a case series further revealed neurological symptoms and radiographic findings associated with SARS-CoV-2 infection in children (4), though they were reported to less likely become severely ill than older adults. These reports jointly emphasized the neurological involvement in COVID-19 patients. Of note, current studies on the neurology of hospitalized COVID-19 patients almost relied on the reverse transcription-polymerase chain reaction (RT-PCR) for detection of SARS-CoV-2 presence using CSF as the available CNS sample. Given these manifestations resulted from the direct invasion of SARS-CoV-2 into CNS, however, the SARS-CoV-2 detection rate in CSF was quite inconsistent and most studies of high quality failed to validate the presence (3, 5). It was possible that the traditional RT-PCR was not sensitive enough in CSF sample. More direct evidence came from an autopsy study of brain tissues from 18 deceased patients, in which RT-PCR for the SARS-CoV-2 nucleocapsid protein was only positive in few sections with the remaining negative or equivocal (6).

**Figure 1:**
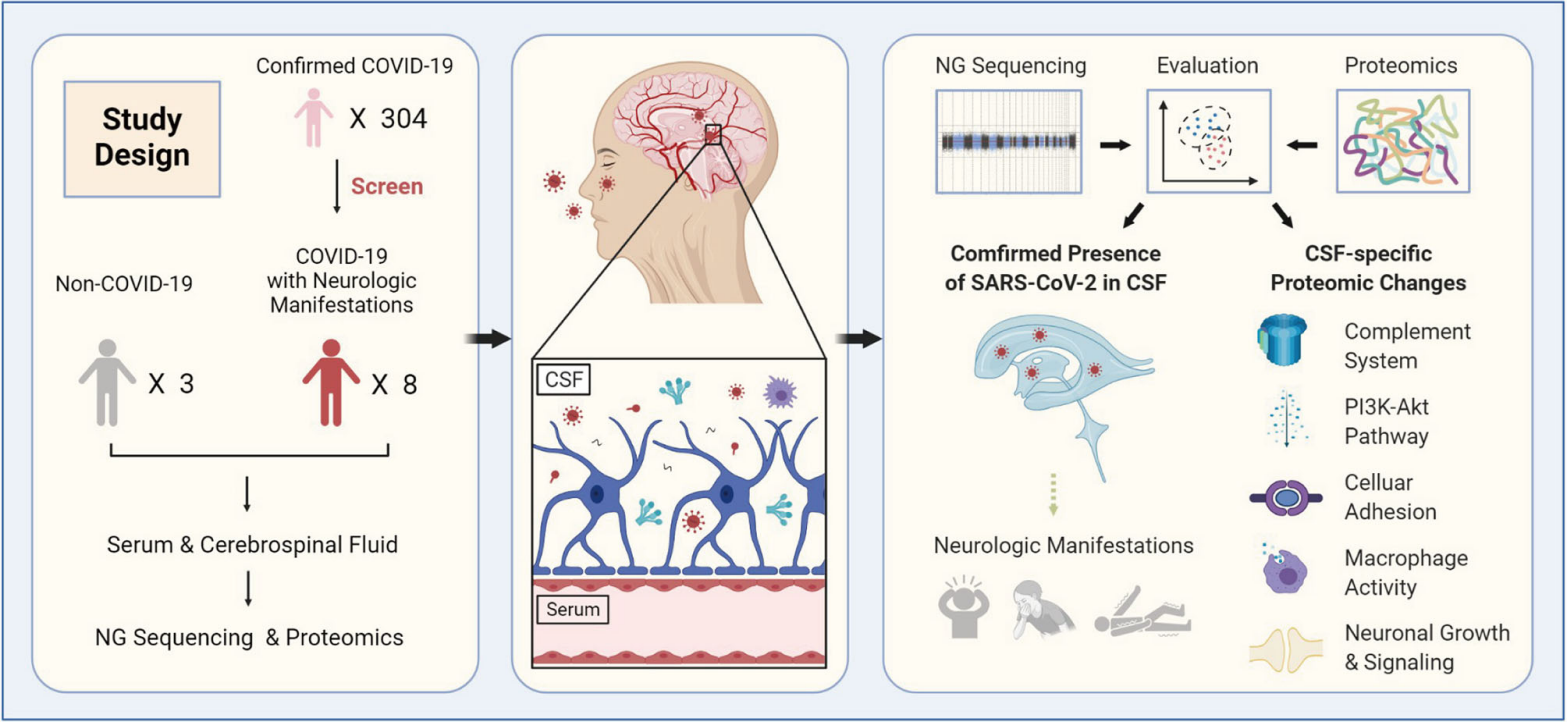
Graphic abstract. 304 COVID-19 in-hospital patients were enrolled in this study to screen out those with prominent new-onset neurological manifestations. Finally, 8 COVID-19 patients with neurological manifestations and three non-COVID-19 patients were selected. NGS and proteomics analysis were conducted in the simultaneously obtained CSF and serum samples from the selected patients. SARS-CoV-2 RNA were validated to present in the CSF of COVID-19 patients, providing the direct clue that SARS-CoV-2 may invade central nervous system. The proteomic results highlighted the involvement of complement system, PI3K-Akt signaling pathway, enhanced cellular interaction, and macrophages in pathogenesis of the COVID-19 related neurological manifestations.

Routine microbiologic testing may be insufficient to detect SARS-CoV-2 in CNS sample. Next-generation sequencing (NGS) is a promising approach for the diagnosis of CNS infection with a comprehensive spectrum, especially for emerging infections. Proteomic-based approach has been widely applied in exploring comprehensive changes on protein level and no proteomics of CSF from COVID-19 patients has been reported. Here, we employed NGS and proteomics to investigate the CSF from COVID-19 patients with neurological manifestations, with the aim to validate the CNS infection of SARS-CoV-2 and elucidate the corresponding proteomic changes of CSF (**Figure 1**).

## Materials and Methods

### Participants and Data Collection

Eight COVID-19 patients with neurological manifestations, who were screened out of 304 COVID-19 in-hospital patients, and three non-COVID-19 patients at Wuhan Red-Cross Hospital from January 24 to March 17, 2020 were enrolled in this study. All the eight patients were diagnosed as COVID-19 by RT-PCR-positive nasopharyngeal or oropharyngeal swabs as described previously (7). This study had been approved by the Institutional Ethics Board of Wuhan Red-Cross Hospital. Written consent from all patients was obtained and filed. Clinical data were collected from electronic medical records.

### CSF and Serum Sample Collection

CSF was obtained through a lumbar puncture. Briefly, a needle was inserted between the 3^rd^ and 4^th^ lumbar vertebrae, and 10 ml CSF was collected into a viral-protecting solution. 5 ml blood was collected by vein puncture and serum was obtained after centrifugation at 1,000 g, 4°C for 15 min. All the samples were stored at -20°C until NGS and proteomic analysis.

### Sample Preparation and Liquid Chromatography-Tandem Mass Spectrometry (LC-MS/MS) Analysis

Ten microliters (μL) of serum were mixed with 190 μL reaction solution, followed by incubation at 60°C for 30 min for sample inactivation, protein denaturation, disulfide bond reduction, and cysteine-SH alkylation. Protein concentration was measured and samples were diluted with equal volume of H_2_O to bring SDC concentration down to 0.5% for tryptic digestion. Trypsin was added at a ratio of 1:50 (enzyme: protein, w/w) for overnight digestion at 37°C. After centrifugation (12,000×g, 15 min), supernatant was subjected to peptide purification using self-made desalting columns (8). Peptide elute was vacuum dried and stored at -20°C for later use.

The CSF samples were centrifuged to remove red-blood cells and then were heat-inactivated at 56°C for 30 min. Protein was precipitated and the pellet was dissolved in a buffer consisting of 8 M Urea in 100 mM Tris-HCl (pH 8.0). The supernatant was used for reduction reaction, and followed by alkylation reaction. Protein concentration was measured by Bradford method. Urea was diluted below 2 M using 100 mM Tris-HCl (pH 8.0). Trypsin was added at a ratio of 1:50 for overnight digestion at 37°C. Next day, trifluoroacetic acid (TFA) was used to bring the pH down to 6.0 to end the digestion. After centrifugation, supernatant was subjected to peptide purification, followed by lyophilization. TMT labeling was performed according to manufacturer’s instructions of the TMT kit (Thermo Fisher Scientific Inc.).

LC-MS/MS data acquisition was carried out on a Q Exactive HF-X mass spectrometer coupled with an Easy-nLC 1200 systems previously reported (9).

### Next-Generation Sequencing (NGS) or RNA-Seq

Total RNA was extracted from 4.5 ml CSF from each patient using TRlZOL Reagent (Ambion^®^) (invitrogen,15596-018). The RNA was further purified with two phenol-chloroform extractions, and dissolved in 22 µl RNA-free water. The quality and quantity of the purified RNA were determined by measuring the absorbance at 260nm/280nm (A260/A280) using NanoDrop 2000 (Thermo). RNA integrity was further verified by 1.5% agarose gel electrophoresis.

For each sample, 10 μl total RNA was used for RNA-seq library preparation. Ribosomal RNAs were depleted with Ribo-off ™ rRNA depletion kit (Vazyme, N406-01). The purified RNA was treated with RQ1 DNase (Promega, M610A) to remove DNA before preparing the directional RNA-seq library by KAPA Stranded mRNA-Seq Kit for Illumina^®^ Platforms (Roche, KK8544). Polyadenylated mRNAs were purified and Fragmented. Fragmented mRNAs were converted into double stranded cDNA. Following end repair and A tailing, the DNAs were ligated to Diluted Roche Adaptor. After purification of ligation product and amplification, the size fraction of 300-500 nt, was purified, quantified and stored at -80°C before sequencing. The strand marked with dUTP (the 2nd cDNA strand) was not amplified, allowing strand-specific sequencing.

For high-throughput sequencing, cDNA libraries were applied on Illunima Novaseq 6000 system using 150 nt paired-end kit for sequencing. The RNA extraction and library preparation service were provided by ABLife (Wuhan, China), and the sequencing service was provided by Novogene (Tianjin, China).

### Data Analysis and Statistics

MS raw data were analyzed with MaxQuant (V1.6.6) using the Andromeda database search algorithm. Spectra files were searched against the UniProt human protein database and NCBI SARS-CoV-2 protein database. MS1 match tolerance was set as 20 ppm for the first search and 4.5 ppm for the main search; MS2 tolerance was set as 20 ppm. Search results were filtered with 1% false discovery rate (FDR) at both protein and peptide levels.

For categorical variables, the data were expressed as frequency or percentage (%). For continuous variables, the data with a normal distribution were expressed as mean ± standard deviation (SD), while those without a normal distribution, expressed as median (IQR). Means for continuous variables were compared using independent group two-tailed t-tests when the data were normally distributed. All statistical analyses were performed using SPSS 20.0 software, and *P*<0.05 was regarded as statistically significant.

## Results

### Demographic and Clinical Characteristics

**Figure 2:**
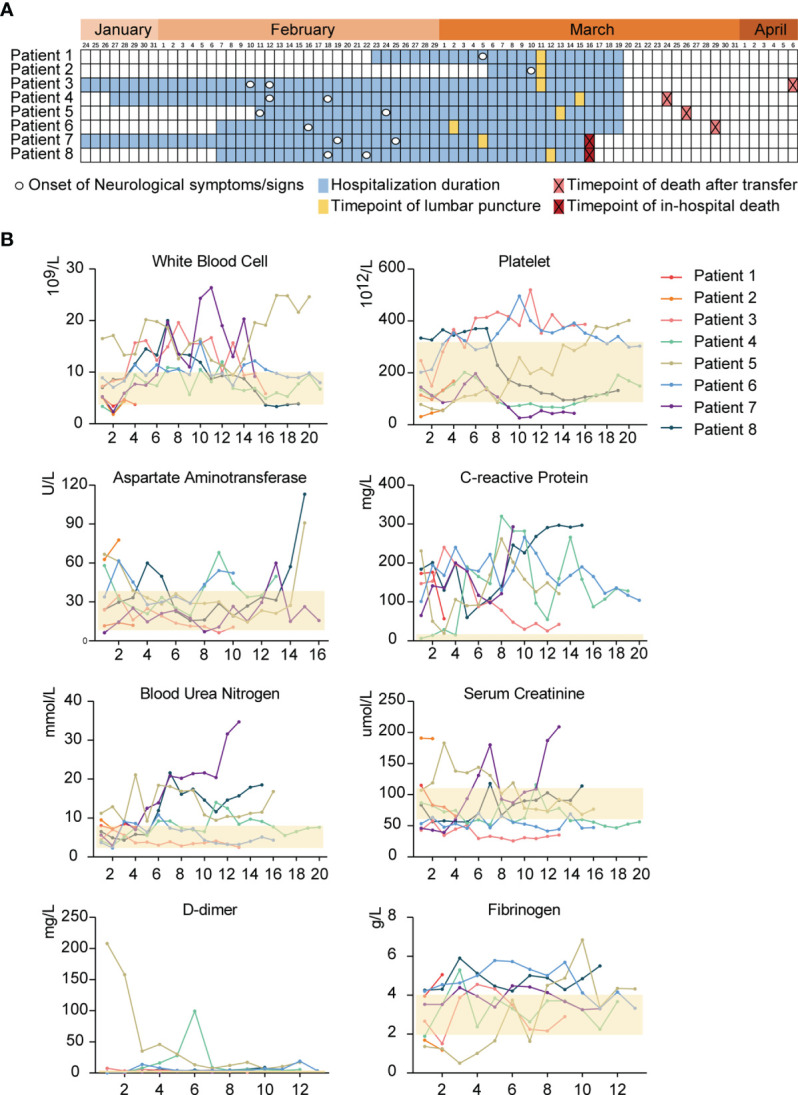
The timeline of clinical events engaged in eight COVID-19 patients and the consecutive routine tests. **(A)**, The illustration summaries the hospitalization duration, the onset time of neurologic symptoms/signs, the time-points of lumbar puncture, and the outcomes of 8 COVID-19 patients with neurological manifestations. **(B)**, Continuous routine tests about hemogram, enzymonram and coagulation for 8 COVID-19 patients. The semitransparent yellow rectangles represent the normal ranges for different parameters. The horizontal ordinates all indicate the times of tests after admission.

To improve the positive rate in detecting the potential SARS-CoV-2 CNS infection, eight patients with neurological manifestations were screened out from 304 COVID-19 patients whose SARS-CoV-2 infection was confirmed by pharyngeal swab RT-PCR tests (**Figure 1**). For the eight patients, the median age was 65.5 years old and two of them were female. Seven elderly patients, with age ranging from 58 to 90 years-old, had different underlying diseases; and among them, one had a history of psychiatric disorders without relapse. The initial symptoms for their COVID-19 included cough, sputum, fever, dyspnea, fatigue, rigor, headache, dizziness, diarrhea, and stomachache. Among these eight COVID-19 patients, six developed into critically ill cases and were admitted to the Intensive Care Unit (**Table 1**). Their median hospitalization duration was 40.5 days (IQR, 35-53) (**Table 1** and **Figure 2A**). After their admission, routine tests including hemogram, enzymonram and coagulation were conducted regularly. All the patients showed certain abnormalities in these parameters during their hospitalization, among which leukocytosis and the elevation of C-reactive protein and D-dimer were the most prominent (**Figure 2B**). For these eight patients, two recovered and were discharged while the other six patients eventually died from respiratory and circulatory failure, multiple organ failure, or septic shock (**Table 1**).

**Table 1:** Demographic and clinical features of patients.

	Patient 1	Patient 2	Patient 3	Patient 4	Patient 5	Patient 6	Patient 7	Patient 8
**Sex/age, years old**	Male/66	Male/58	Male/31	Male/62	Male/67	Female/80	Male/65	Female/90
**Underlying disease**	Hypertension, Diabetes, Chronic bronchitis, Arterial fibrillation, Psychiatric disorder^a^	Hepatitis B, Hepatitis C, Cirrhosis	None	None	Perinephritis	Hypertension	Chronic gastritis, Post-esophagectomy	Hypertension
**Initial Symptoms**	Cough, Sputum, Dyspnea	Fever, Diarrhea, Stomachache	Fever	Fever, Cough, Fatigue, Headache, Dizziness	Cough, Dyspnea	Fever, Nonproductive cough	Fever, Cough, Sputum	Fever, Rigor
**Neurological symptoms/signs (days after admission)**	Psychiatric disorder (12)	Headache (5), Mania (5)	Headache (18), Stiff-neck (20)	Light coma (26), Stiff-neck (20)	Light coma, Uroclepsia (1), Stiff-neck (14)	Medium coma with weak PLR^e^ (10)	Medium coma with weak PLR (27), Epilepsy (33)	Medium coma with weak PLR (16), Stiff-neck (12)
**Respiratory support**	No	No	Tracheotomy, Invasive mechanical ventilation	Endotracheal intubation, Invasive mechanical ventilation	Tracheotomy, Invasive mechanical ventilation	Endotracheal intubation, Invasive mechanical ventilation	Endotracheal intubation, Invasive mechanical ventilation	Endotracheal intubation, Invasive mechanical ventilation
**SARS-CoV-2 in CSF by NGS**	Suspicious^b^	Suspicious^b^	Detected	Detected	Suspicious^b^	Suspicious^b^	Detected	Detected
**CSF IgM for** **SARS-CoV-2 (AU/mL)** ^c^	0.06	0.07	0.06	0.09	0.06	0.08	0.10	0.05
**CSF IgG for** **SARS-CoV-2 (AU/mL)** ^c^	0.52	0.65	0.07	2.34	0.54	1.58	4.83	0.81
**Five other Virusesin CSF by NGS** ^d^	Not detected	Not detected	Not detected	Not detected	Not detected	Not detected	Not detected	Not detected
**SARS-CoV-2 in Serum by NGS**	Negative	Negative	Negative	Negative	Negative	Negative	Negative	Negative
**Transfer to ICU**	No	No	Yes	Yes	Yes	Yes	Yes	Yes
**Outcome**	Discharged	Discharged	Death	Death	Death	Death	Death	Death
**Cause of Death**	–	–	Respiratory and circulatory failure	Multiple organ failure	Respiratory and circulatory failure	Multiple organ failure	Multiple organ failure	Septic shock, Multiple organ failure

a Long-term clinical remission.

b Viral read clusters were located only at 3’terminal of the genome of SARS-CoV-2.

c Negative when <10 AU/mL.

d Five other Viruses: Poliovirus, Coxsackie virus, ECHO virus, Adenovirus, Herpes simplex virus.

e PLR, pupillary light reflex, same below.

### Neurological Manifestations, CSF Examination, and Cerebral CT Scan

All the eight COVID-19 patients presented with different neurological symptoms/signs during hospitalization, including coma, sluggish pupillary light reflex, headache, acute encephalopathy, epilepsy, stiff-neck (meningeal signs), and psychiatric disorder. Cerebral infarction was found in one patient by Computed Tomography (CT) (**Figure S1**). The period from admission to the onset of neurological symptoms/signs ranged from 1 to 33 days (**Figure 2A**). Diagnostic lumbar puncture was performed for all the eight patients. Seven of the CSF samples showed a clear colorless appearance, with a normal range of white blood cells (WBCs) ([4-8] x 10^6^/L) and slightly increased proteins (0.5-0.7 g/L). One CSF sample showed slight turbid color as well as increased WBCs (80 x 10^6^/L). Although neurological manifestations may stem from a variety of reasons, SARS-CoV-2 was speculated to present in the CNS and might at partially account for these disorders.

### Virus Detection in CSF Samples

The reports on the presence of SARS-CoV-2 in the CNS of COVID-19 patients was quite inconsistent. Some studies failed to detect SARS-CoV-2 in CSF by RT-PCR despite the prominent neurological manifestations (3, 5). This might be due to limited sensitivity of conventional laboratory testing method or low viral titer in the CSF samples. Metagenomic Next Generation Sequencing (mNGS), as a transformative high-throughput approach to the diagnosis of infectious diseases, has the merit to detect a comprehensive spectrum of potential pathogen genomes. The power of NGS has been demonstrated in the diagnosis of CNS infections such as meningitis and encephalitis (10). Therefore, we decided to conduct metagenomic sequencing of the CSF samples from the eight COVID-19 patients, together with serum samples simultaneously obtained from the same patients as controls.

**Figure 3:**
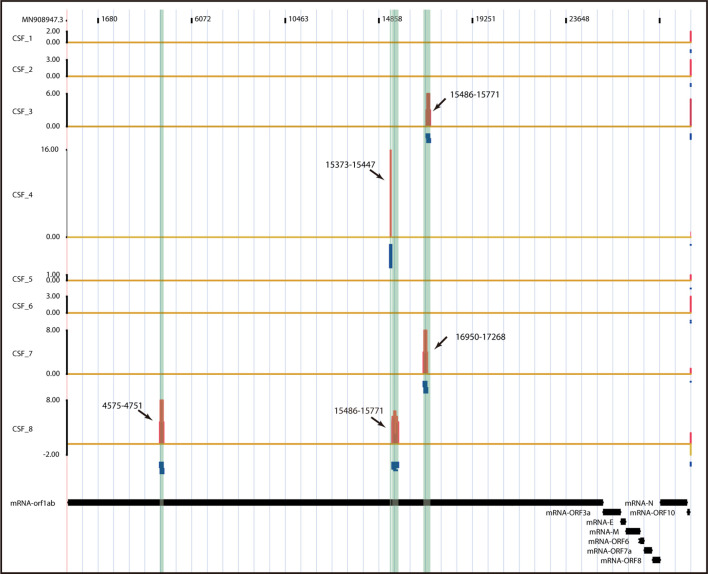
Visualization of SARS-CoV-2 RNA-Seq Read. For each CSF sample, total RNA was extracted and used for RNA-seq library preparation after the rRNA removal. Then the cDNA libraries were applied to Illunima Novaseq 6000 system for 150 nt paired-end sequencing. Reads unique to SARS-CoV-2 were mapped on the SARS-CoV-2 genome. The chart illustrates the distribution of SARS-CoV-2-specific reads on SARS-CoV-2 genome from the eight CSF samples in histograms (red) and read clusters (blue).

SARS-CoV-2 RNA sequences were indeed detected unequivocally in the CSF samples from four patients, and CSF samples of the four remaining patients were deemed likely to contain SARS-CoV-2 RNA sequences (**Figure 3**). The SARS-CoV-2 IgM and IgG antibodies were negative in all of the eight CSF samples (**Table 1**). Interestingly, SARS-CoV-2 RNA was negative in all of the eight serum samples, possibly due to the low titer of viral RNA in serum or the neutralization by SARS-CoV-2 specific antibodies in the time-point of our sample collection. Consistently, both IgG and IgM specific for SARS-CoV-2 were positive in the sera of all the eight patients. These data indicate that SARS-CoV-2 can be present in the CNS even after the virus has been cleared from peripheral blood.

### Proteomic Alterations in CSF

After finding SARS-CoV-2 in CSF samples, we further investigated the proteomic changes of CSF in an effort to infer the mechanisms of SARS-CoV-2 infection in the CNS. Taking into consideration that the blood-brain barrier (BBB) permeabilization may have been altered in the CNS of COVID-19 patients, which could impact the systemic inflammation in serum and vice versa, we conducted a quantitative proteomic analysis of the CSF samples, along with simultaneously obtained serum samples, from COVID-19 patients (COVID-19 group, n = 8) *versus* non-COVID-19 patients (control group, n = 3) from the same hospital.

**Figure 4:**
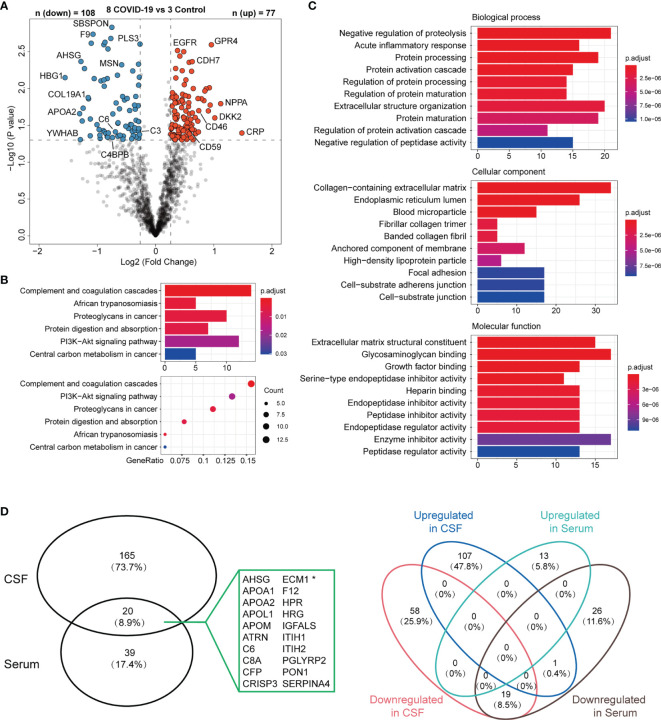
DEPs of CSF from 8 COVID-19 patients with Neurologic Manifestations vs Control patients. **(A)**, Volcano plot for identified proteins in CSF between 8 COVID-19 patients and 3 control patients. Proteins with fold-change beyond 1.2 or below -1.2 with p value lower than 0.05 were considered as significantly differential expression. **(B, C)** KEGG Enrichment Pathway and GO Oncology analysis of differentially expressed proteins with significance (n =185) that corresponded in the **(A)**. **(D)** The diagram shows 185 proteins in CSF and 59 in serum were significantly altered in COVID-19 vs control groups. Note that only 20 proteins were in common, indicating the limited overlap. Venn diagrams showing that there were 20 proteins shared between CSF DEPs and Serum DEPs. 19 shared proteins were consistently downregulated in both samples while one protein (marked with asterisk in A) showed the opposite change in two samples.

In the CSF samples, there were 108 up-regulated and 77 down-regulated proteins in the COVID-19 group, compared with the control group. Among the DEPs in the CSF, the prominent marker C-reactive protein (CRP) was increased with the highest fold change (**Figure 4A**), indicating an active inflammation in the CNS. In serum samples, 59 proteins were significantly altered in COVID-19 patients compared to the control group, with 13 up-regulated and 46 down-regulated. Among these differentially expressed proteins (DEPs) from CSF vs serum samples, only 20 proteins were in common and were all down-regulated except ECM1 showed upregulation in CSF (**Figure 4D**).

**Figure 5:**
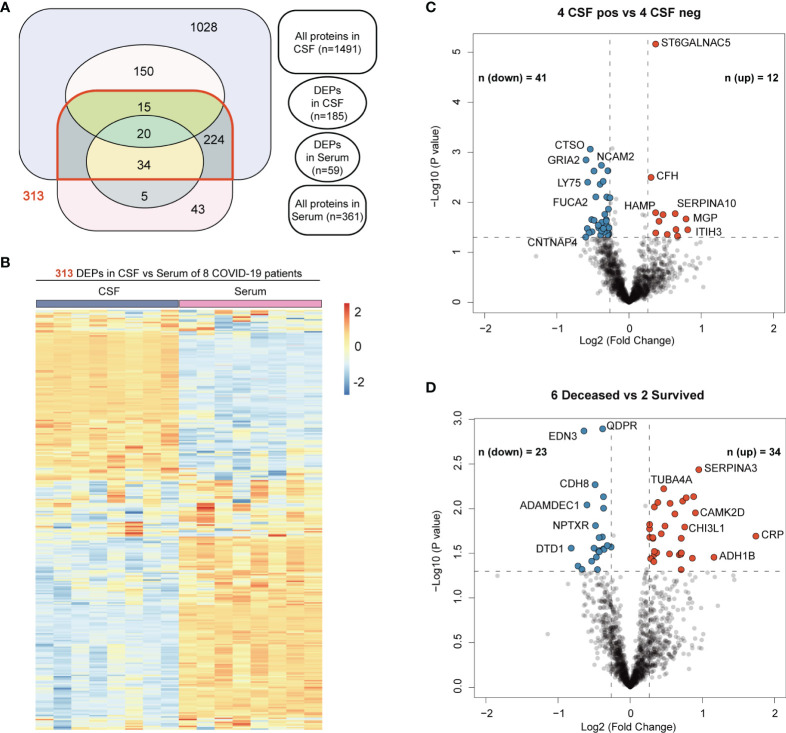
The correlation between the original protein expression profile of CSF and serum samples in 8 COVID-19 patients and Volcano Plots relevant to different subgroup comparisons. **(A)** Venn diagrams showing the number of all proteins identified and quantified by proteomics in CSF and serum of 8 COVID-19 patients and their intersections with DEPs generated in different comparison patterns. **(B)** Heatmaps of expression levels of 313 proteins shared by CSF and serum proteomic profile. **(C)** Volcano plot for identified proteins in CSF between CSF SARS-CoV-2 positive (4) and negative (4) patients. **(D)** Volcano plot for identified proteins in CSF between deceased (6) and survived (2) patients. Proteins with fold-change beyond 1.2 or below -1.2 with p value lower than 0.05 were considered as significantly differential expression.

We further checked the correlation between the original protein expression profile of CSF and serum samples in 8 COVID-19 patients. There were 1491 and 361 proteins unbiasedly identified and quantified in the CSF and serum samples, respectively. Surprisingly, about four times as many proteins were detected in CSF compared to serum. This contrasting protein abundance may reflect more active perturbation occurred in central nervous system and were reminiscent of the prominent neurological manifestations in these patients. 313 proteins were shared by these two samples, which accounted for 20.1% (313/1491) of total proteins in CSF (**Figure 5A**). As illustrated by heatmap, the expression level of these shared proteins also presented great polarity in the two sets of samples (**Figure 5B**). Altogether, these data suggested that low correlation exited in the proteomic profiles of the CSF and serum samples from COVID-19 patients and most of the DEPs in the CSF were not shared by peripheral serum samples. Therefore, SARS-CoV-2 invasion of the CNS resulted in CSF-specific proteomic changes, suggesting that the neurological manifestations might be caused by local SARS-CoV-2 infection in the CNS, rather than by systemic inflammation. Further analysis of the DEPs in CSF may provide insight into the pathogenesis in the CNS of COVID-19 patients.

### Functional Classification of Differentially Expressed Proteins in CSF

**Figure 6:**
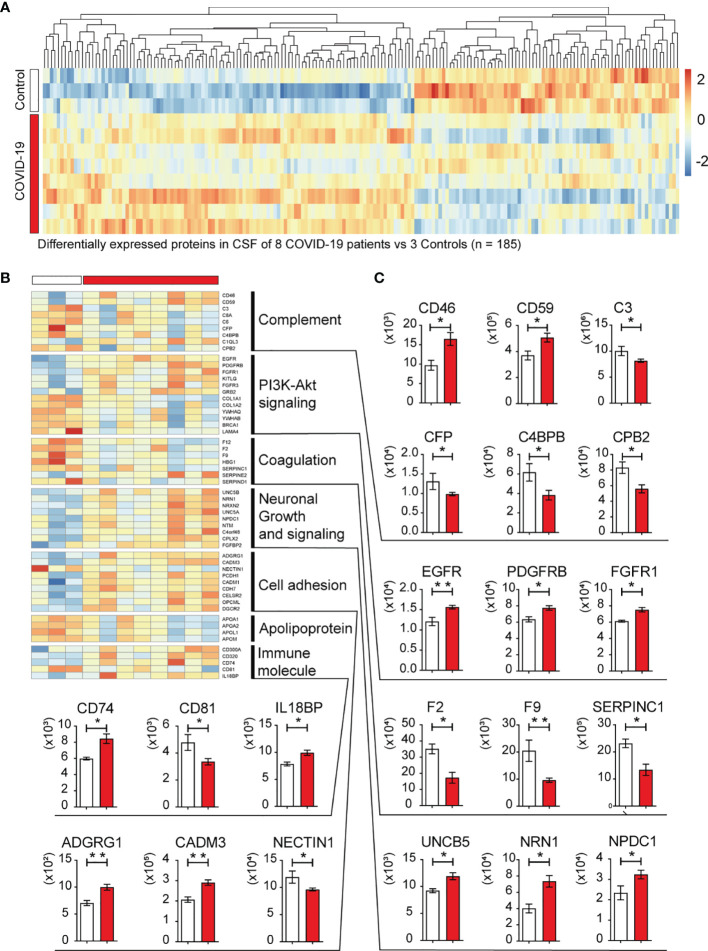
Heatmap of 185 DEPs in CSF and expression values of selected DEPs. **(A)** Heatmap of significant DEPs (n=185) in CSF of eight COVID-19 patients vs three non-COVID-19 control patients. **(B)** Heatmap of selected DEPs enriched in KEGG pathway analysis or found to be important after manual inspection. **(C)** The expression level (Original value) of representative DEPs in CSF between COVID-19 and control cases. o; *P<0.05, and **P<0.001 by two-tailed unpaired Student t test.

KEGG pathway and Gene Oncology analysis were performed in order to integrate the DEPs into multiple biological processes potentially involved in the pathogenesis of neurological disorders. The analysis revealed that the highest proportion of DEPs (14 proteins or 13.7%) were those involved in complement and coagulation cascades (**Figure 4B**), including membrane cofactor protein (MCP; CD46), membrane attack complex inhibitory protein (CD59), complement C1q-like protein 3 (C1QL3), complement C4 binding protein B (C4BPB), carboxypeptidase B2 (CPB2), complement C3, C6, and C8a (**Figures 4A** and **6**). And the hyperactivation of complement system has been reported to a distinctive feature of severe COVID-19 patients (11, 12). The PI3K-Akt signaling pathway was the second most affected pathway, with 12 DEPs identified by the KEGG pathway analysis (**Figures 4B** and **6**). Consistently, a PI3K/AKT signal pathway inhibitor capivasertib has been confirmed to restrict the entry of SARS-CoV-2 into cells under non-cytotoxic concentrations (13). The Gene Oncology analysis further revealed the infected neural microenvironment probably experienced intensive protein turnover, and these proteins were widely connected in the different cellular compartments, including extracellular matrix, membrane, and endoplasmic reticulum (**Figure 4C**). For further comprehensive understanding of resuts, manual inspection of other DEPs in reference to the UniProt database allowed for assigning them into several functional clusters, belonging to pathways such as neuronal growth and signaling, and cell adhesion (**Figures 6B, C**). These differentially expressed molecules well reflected the injury of neurons and the altered cellular interactions in the CNS invaded by SARS-CoV-2. Apolipoproteins APOA1, APOA2, APOL1, and APOM, which have been previously reported to be associated with macrophage and decreased in COVID-19 patients (14), were all downregulated in the CSF from the eight COVID-19 patients. Thus, the perturbation of complement system, PI3K-Akt signaling pathway, neuronal growth and signaling, cell adhesion, and macrophage may jointly underlie the pathogenesis of the SARS-CoV-2 infected CNS and neurological manifestations in COVID-19 patients.

To investigate the underling differences of CSF proteomics profiles amongst these 8 COVID-19 patients, we conducted subgroup comparison based on the NGS results and their clinical outcomes (**Table 1**). As expected, there exited less DEPs (**Figures 5C, D**) and less KEGG enrichment pathway engaged. The Gene Oncology analysis also yielded less results with statistically significance (**Figure S2**). The main difference of CSF between patients with different NGS results may be implicated in the lysosome-related proteins. As mentioned above, the marker CRP also present highest fold change in the CSF between the 6 deceased and 2 survived patients, which indicated its potential to predict the prognosis (**Figure 5D**).

## Discussion

We took advantage of broad spectrum and sensitivity of NGS to detect SARS-CoV-2 in the CSF of COVID-19 patients. This study provides convincing and direct evidence for the presence of SARS-CoV-2 in the CNS (15). Our study has further identified CSF-specific proteomic alterations in COVID-19 patients with neurological manifestations.

NGS is a sensitive method for detecting neuro-invasive viral pathogens in the CNS. Indeed, a study of 58 COVID-19 patients with encephalopathy and signs of corticospinal tract damage failed to detect the virus in the cerebrospinal fluid using RT-PCR (3). In our current study, we confirmed the presence of SARS-CoV-2 in CSF obtained from COVID-19 patients by NGS, suggesting that high-throughput sequencing may be more sensitive than RT-PCR for pathogenic detection in CSF.

It was presumed that SARS-CoV-2 can gain access to the CNS through two distinct routes, namely the hematogenous and transneural routes. The former involves either directly infecting the endothelial cells of the BBB or taking a ride on leukocytes that cross the BBB (the ‘‘Trojan Horse’’). The later route involves the olfactory nerve associated with the nasal epithelium. The olfactory bulb may serve as the transneural route for Human CoVs to reach the CNS. Sungnak et al. have demonstrated high expression of viral entry factors (e.g. ACE2) in nasal epithelial cells of healthy human tissues (16), suggesting that olfactory bulb may serve as a potential portal for viral neuro-invasion.

Although we detected the SARS-CoV-2 viral RNA in CSF samples by NGS, SARS-CoV-2 IgM and IgG were not found in the same samples. This might be due to the immune privilege of CSF, where efficient adaptive immune response is lacking. It should be noted that, however, in all the serum samples simultaneously obtained from the same patients, we consistently detected either IgM or IgG, although they were negative for SARS-CoV-2 viral RNA. It might be that serum SARS-CoV-2 viral RNA had been neutralized due to the presence of SARS-CoV-2 specific antibodies by the time of sample collection. Given that our investigation was performed at relative late stages of COVID-19 for the six severe cases (patient 3 to 8) and the non-severe condition (patients 1 and 2), the titer of viral RNA in patients’ serum might already be too low to be detected. Another possibility still worthy of consideration is that the CNS infection in these patients might be independent of their peripheral blood viral infection and thus might not have happened simultaneously with viremia.

SARS-COV-2 is known to infect and damage endothelial cells (17), the major cellular component of the BBB. However, the comparison of proteomics obtained from CSF vs serum suggested that these two sets of samples displayed significantly distinct characteristics, with only a small percentage of changes shared. In CSF, complement activation was prominent, with a dramatic up-regulation of the complement regulatory protein CD46. Of note, CD46 can serve as the receptor for a number of viruses and can modulate adaptive immune responses (18). Whether CD46 can also be exploited by SARS-CoV-2 to infect cells remains worthy of study. The proteomics study also highlights the involvement of PI3K-Akt signaling pathway, neuronal growth and signaling, cell adhesion, and macrophage in SARS-CoV-2 induced pathogenesis in the CNS. Some of them have also been validated in other study about SARS-CoV-2 (13, 14, 19, 20). As tailed therapy for COVID-19 related neuropathogenesis is lacking, targeting these pathways probably affords some clinical benefits.

This study has several limitations. First, the sample size was small because the number of COVID-19 patients dramatically declined in mid-March, 2020 in Wuhan, China. Second, due to the unlikelihood to have real normal CSF control samples, we used only three non-COVID-19 patients who had diagnostic lumbar puncture at the same time frame as the control group, and more control patients should have been included if possible. Third, owing to extremely limited clinical resources in the COVID-19 outbreak then, we were unable to do more specialized neurologic tests for these patients, such as cerebrospinal fluid autoantibodies analysis, brain MRI, and we also failed to obtain autopsy from deceased patients. Further research about the mechanism by which SARS-CoV-2 gained entrance to the CNS and in larger scale is needed.

## Data Availability Statement

The proteomics data presented in the study are deposited in the ProteomeXchange Consortium (https://www.iprox.org/), accession number IPX0003711000. The NGS data presented in the study are deposited in the Sequence Read Archive, accession number PRJNA780093.

## Ethics Statement

The studies involving human participants were reviewed and approved by Wuhan Red Cross Hospital, Wuhan, Hubei, China. The patients/participants provided their written informed consent to participate in this study. Written informed consent was obtained from the individual(s) for the publication of any potentially identifiable images or data included in this article.

## Author Contributions

DH, NX, and SL had roles in the study design, clinical management. HW, ZZ, JZ, SL, SH, YN, and ZK collected and analyzed data. ZZ, AS, and WL drafted the manuscript. HC, HF, HZ, ZL, AS, WL, JL, and SL interpreted the data. DH, SL and NX are responsible for summarizing all data. All authors contributed to the article and approved the submitted version.

## Funding

This study was funded by the National Natural Science Foundation of China (82161138003, 31770983, 82070136, 81201026), the Natural Science Foundation of Hubei Province (2020BHB016), the Russian Foundation for Basic Research (21-51-55003), and by the Postdoctoral Science Foundation of China (2020T130040ZX).

## Conflict of Interest

The authors declare that the research was conducted in the absence of any commercial or financial relationships that could be construed as a potential conflict of interest.

## Publisher’s Note

All claims expressed in this article are solely those of the authors and do not necessarily represent those of their affiliated organizations, or those of the publisher, the editors and the reviewers. Any product that may be evaluated in this article, or claim that may be made by its manufacturer, is not guaranteed or endorsed by the publisher.

## Supplementary Material

Supplementary Figure 1 The head Computed Tomography (CT) Images of the patient with new onset cerebral infarction. Patient 5, Cerebral infarction in right temporal occipital lobe (green), and cerebral infarction with hemorrhage (red) in left occipital lobe were detected by computed tomography (CT).

Supplementary Figure 2 Heatmaps, KEGG enrichment pathway and Gene Oncology analysis of DEPs in CSF generated in different subgroup comparisons. **(A–C)** Heatmaps, KEGG enrichment pathway and Gene Oncology analysis of DEPs between CSF SARS-CoV-2 positive (n = 4) and negative (n = 4) patients. **(D–F)** Heatmaps, KEGG enrichment pathway and Gene Oncology analysis of DEPs between survived (n = 2) and deceased (n = 6) patients.

Supplementary Figure 3 Pearson correlation analysis of 313 shared proteins in CSF and serum of 8 COVID-19 patients. Pearson correlation analysis for the expression values of 313 proteins shared in CSF and serum of 8 COVID-19 patients. Pearson correlation coefficient (R) and the P value were calculated. 6 non-immunoglobulin proteins were screened out with 0.001 as the threshold for P value, namely, C-reactive protein (CRP), Serum amyloid P-component (APCS), Vitamin D-binding protein (GC), Histidine-rich glycoprotein (HRG), Serum amyloid A-2 protein (SAA2), N-acetylmuramoyl-L-alanine amidase (PGLYRP2).

## Abbreviations

COVID-19, coronavirus disease 2019; SARS-CoV-2, severe acute respiratory syndrome coronavirus-2; CNS, central nervous system; CSF, cerebrospinal fluid; NGS, next-generation sequencing; RNA-seq, RNA sequencing; PLR, pupillary light reflex; DEPs, differentially expressed proteins; RT-PCR, reverse transcription-polymerase chain reaction.
