# Supplementary figures and images for: Next-Generation Sequencing and Proteomics of Cerebrospinal Fluid From COVID-19 Patients With Neurological Manifestations
Source: Front Immunol. 2021 Dec 9;12:782731. doi: 10.3389/fimmu.2021.782731 (PMC8695435; doi:10.3389/fimmu.2021.782731)

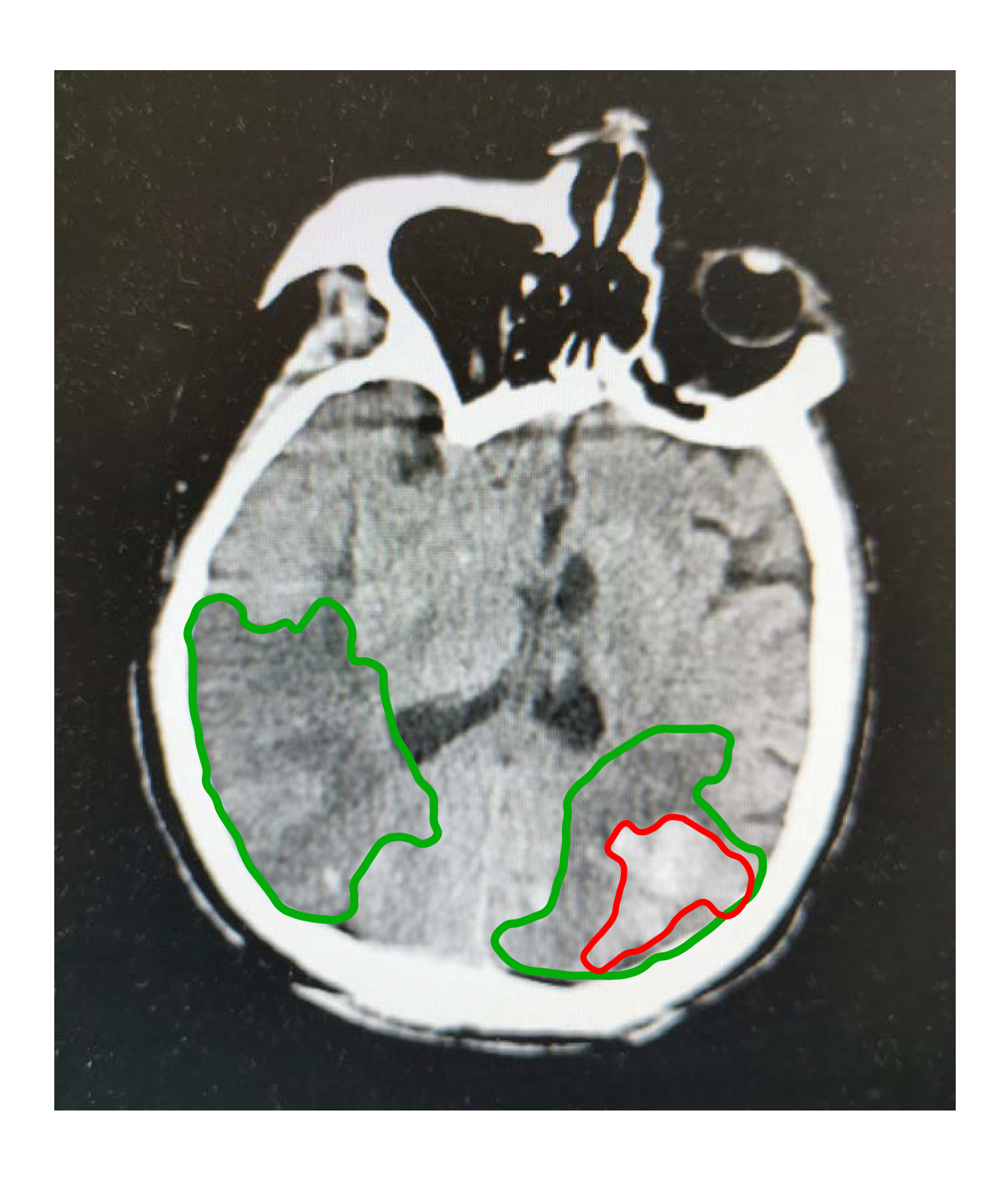

Supplement: Supplementary Figure 1 — The head Computed Tomography (CT) Images of the patient with new onset cerebral infarction. Patient 5, Cerebral infarction in right temporal occipital lobe (green), and cerebral infarction with hemorrhage (red) in left occipital lobe were detected by computed tomography (CT). [file Image_1.tif]

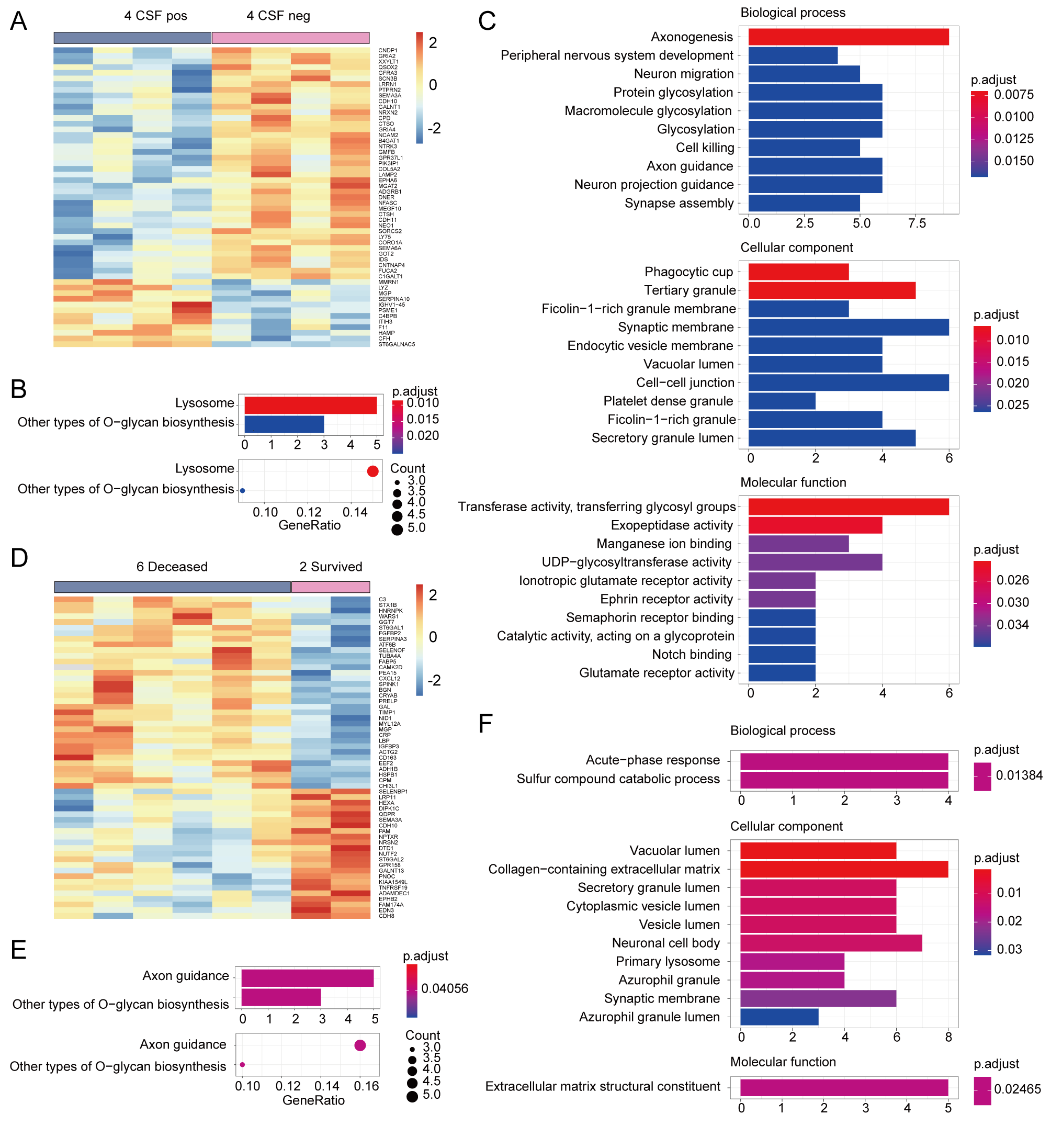

Supplement: Supplementary Figure 2 — Heatmaps, KEGG enrichment pathway and Gene Oncology analysis of DEPs in CSF generated in different subgroup comparisons. (A–C) Heatmaps, KEGG enrichment pathway and Gene Oncology analysis of DEPs between CSF SARS-CoV-2 positive (n = 4) and negative (n = 4) patients. (D–F) Heatmaps, KEGG enrichment pathway and Gene Oncology analysis of DEPs between survived (n = 2) and deceased (n = 6) patients. [file Image_2.tif]

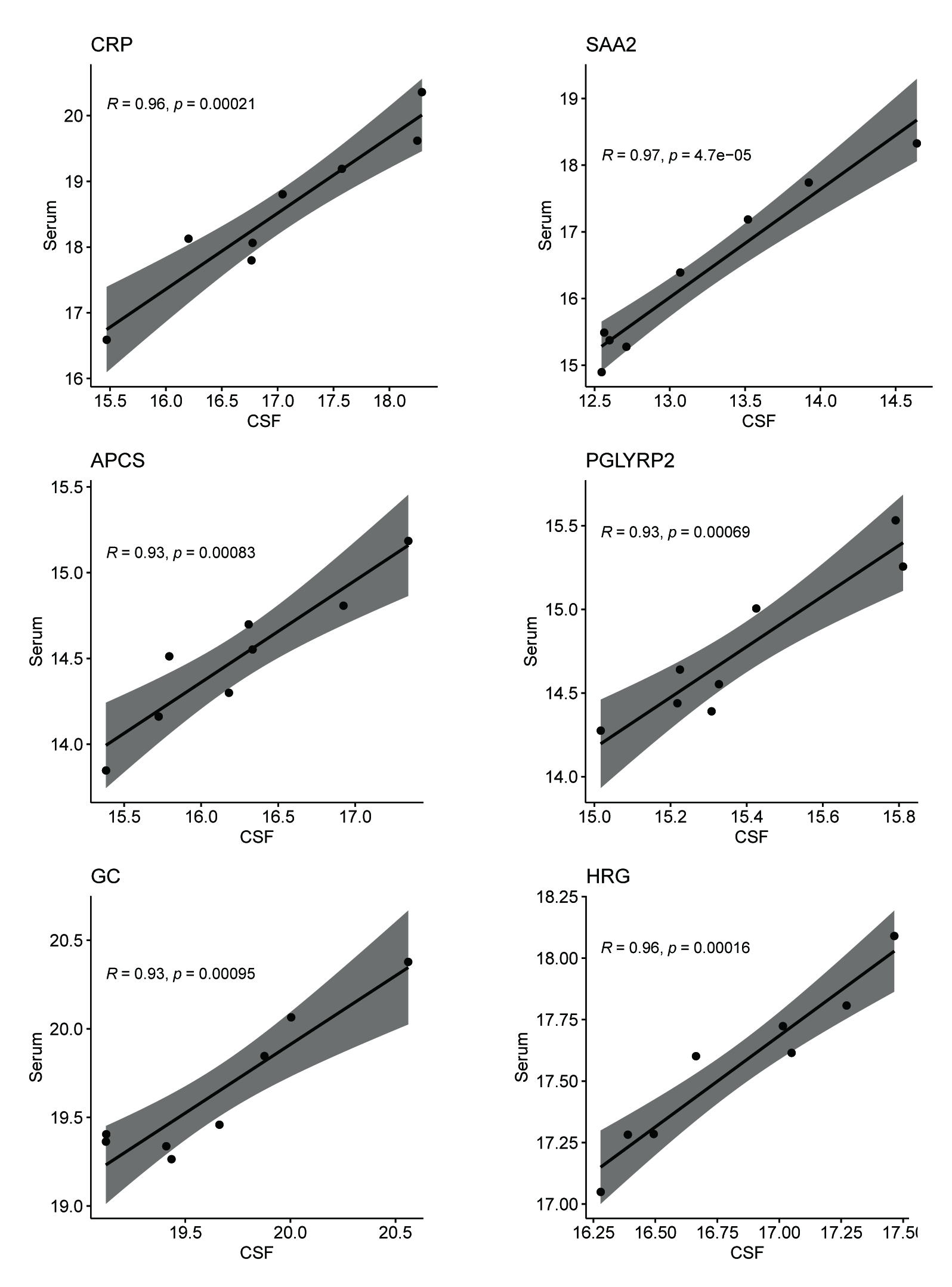

Supplement: Supplementary Figure 3 — Pearson correlation analysis of 313 shared proteins in CSF and serum of 8 COVID-19 patients. Pearson correlation analysis for the expression values of 313 proteins shared in CSF and serum of 8 COVID-19 patients. Pearson correlation coefficient (R) and the P value were calculated. 6 non-immunoglobulin proteins were screened out with 0.001 as the threshold for P value, namely, C-reactive protein (CRP), Serum amyloid P-component (APCS), Vitamin D-binding protein (GC), Histidine-rich glycoprotein (HRG), Serum amyloid A-2 protein (SAA2), N-acetylmuramoyl-L-alanine amidase (PGLYRP2). [file Image_3.tif]
